# Enhancing the response inhibition skill of soccer players with repeated tDCS: a randomized controlled ERP study

**DOI:** 10.3389/fnhum.2026.1738168

**Published:** 2026-03-19

**Authors:** Kaihao Chen, Huan Yu, Shaokun Zhang, Zhikang Li, Jeho Song

**Affiliations:** 1 Department of Sports Science, Wonkwang University, Iksan-si, Jeonbuk State, Republic of Korea; 2 Physical Education Institute, Shangrao Normal University, Shangrao, Jiangxi, China; 3 College of Physical Education and Health, East China Jiaotong University, Nanchang, Jiangxi, China; 4 Physical Education Institute, Jiangxi Normal University, Nanchang, Jiangxi, China

**Keywords:** event-related potential, open-skill sports, response inhibition, soccer player, transcranial direct current stimulation

## Abstract

**Objective:**

This study investigated the effects of repeated transcranial direct current stimulation on response inhibition and sought to elucidate the underlying neurobehavioral mechanisms.

**Methods:**

In a randomized, triple-blind, sham-controlled, 36 male soccer players were assigned to active-tDCS, sham-tDCS, or no-intervention control groups. The active-tDCS group received 20-min 1.5 mA tDCS over the right dorsolateral prefrontal cortex (DLPFC) five times weekly for 4 weeks, alongside regular training. The sham-tDCS group received 1-min 1.5 mA tDCS with regular training, and the no-intervention control group only regular trained. Pre- and post-intervention, all participants performed a Go/No-go task while behavioral and event-related potential (ERP) data were recorded. Behavioral metrics: Go reaction time (RT), Go accuracy (ACC), and No-go accuracy. ERP metrics: P3 amplitude and latency.

**Results:**

Behavioral: Only the active-tDCS group showed significantly shorter Go RT post-intervention compared to baseline and the control group. The ACC for the three groups of Go and No-go tasks remained unchanged. ERP: Only the active-tDCS group exhibited increased P3 amplitude and reduced P3 latency during both Go and No-go trials. A significant three-way interaction indicated that latency shortening in No-go trials was most pronounced at central sites Cz/Cpz. The sham-tDCS group and the no-intervention control group showed no significant changes in P3 amplitude and latency between pre-tests and post-tests.

**Discussion:**

These preliminary findings suggest that repeated tDCS over the right prefrontal cortex may enhance behavioral response speed in soccer players, accompanied by neurophysiological changes indicative of optimized processing efficiency (increased P3 amplitude and shortened latency). However, given the exploratory nature and modest sample size, these results warrant confirmation in larger-scale studies.

**Clinical trial registration:**

https://www.chictr.org.cn/showproj.html?proj=288285, ChiCTR2500109387.

## 1 Introduction

Soccer is an open-skill sport where response inhibition, as a core component of executive function, is crucial (Ali, 2011; Wang et al., 2013). Response inhibition refers to the suppression of behaviors that are no longer needed or are inappropriate in rapidly changing environments (Verbruggen and Logan, 2008). It is crucial for an individual’s motor development, enabling rapid responses to constantly changing movements (Albaladejo-Garcia et al., 2023). For soccer players, possessing exceptional response inhibition enables them to execute tactical maneuvers with precision and immediately rescind the erroneous decision, thereby reducing errors and enhancing athletic performance (Beavan et al., 2019; Albuquerque et al., 2019). Consequently, response inhibition can be used to discriminate between skill levels in soccer players (Verburgh et al., 2014). Research indicates that higher-level soccer players typically exhibit superior executive function, with their response inhibition abilities positively correlated to their performance level in soccer (Vestberg et al., 2012). Traditionally, athletic training has been the primary means of enhancing response inhibition performance (Kao et al., 2022). However, given that cognitive functions are rooted in neural activity within the brain (Blasi et al., 2006), and neural networks involved in response inhibition exhibit significant plasticity (Chambers et al., 2009). This suggests that, in addition to athletic training, neuroscience technologies that regulate brain activity (Roelfsema et al., 2018) may also be effective approaches for enhancing response inhibition.

Transcranial direct current stimulation (tDCS) is a non-invasive brain stimulation technique that modulates cortical excitability via a weak direct current (Fuster, 2008; Nitsche et al., 2008). It was first applied in the field of clinical medicine (Brunoni et al., 2012). Since pioneering research by German scholar Nitsche (Nitsche and Paulus., 2001) in 2001, tDCS has been demonstrated to effectively enhance motor performance (Hummel et al., 2005) and cognitive function (Borducchi et al., 2016). Notably, the underlying mechanism of tDCS involves polarity-specific modulation of cortical excitability. For instance, Carlsen et al. (2015) demonstrated that anodal tDCS typically increases excitability and shortens reaction times in simple tasks, whereas cathodal stimulation decreases excitability and slows responses. The above evidence indicates that tDCS has a positive effect on enhancing participants’ motor performance and cognitive function. However, existing research has primarily focused on investigating the acute effects of a single tDCS session (Stagg et al., 2018). There remains a lack of in-depth exploration into the cumulative benefits of repeated tDCS interventions in athletic populations and the underlying neural mechanisms.

To explore these neural mechanisms, non-invasive tools that directly measure brain activity are essential. Electroencephalography (EEG) (Cohen, 2017), which records electrical activity from the scalp, provides a direct window into the brain’s millisecond-level dynamics with high temporal resolution. The event-related potential (ERP) technique (Luck, 2014), derived from the time-locked averaging of EEG signals, is particularly suited for isolating brain activity associated with specific cognitive processes, such as response inhibition. Over the years, ERP has become one of the most widely used tools for assessing cognitive functions (Sur et al., 2009; Helfrich and Knight, 2019). Distinct ERP components serve as vital windows into studying cognitive functions (Donchin, 2022), providing an objective method for investigating brain activity. Classic ERP components include P1, N1, P2, N2, and P3 (Picton et al., 1995). Among the ERP components elicited by response inhibition tasks, N2 and P3 are regarded as two core indicators (Luijten et al., 2011; Rietdijk et al., 2014; Groom and Cragg, 2015). The N2 component (Patel and Azzam, 2005), a negative wave typically peaking around 200–300 ms post-stimulus, is thought to primarily reflect early conflict monitoring (Rietdijk et al., 2014). The P3 component (Patel and Azzam, 2005), a positive wave usually occurring between 300 and 500 ms after stimulus onset, is one of the most stable and extensively studied endogenous components (Gajewski and Falkenstein, 2013; Groom and Cragg, 2015; Hirao et al., 2020). Evidence suggests that P3 is more closely associated with the successful execution of inhibitory behavior, the late allocation of cognitive resources, and the final evaluation of decision-making (Rietdijk et al., 2014). While both N2 and P3 are implicated in inhibitory function, this study primarily focuses on analyzing the P3 component. The rationale is threefold. First, in sports contexts that demand rapid decision making and action execution, such as football, the later implementation stage of inhibitory control (P3) is postulated to have a more direct contribution to behavioral outcomes compared to the earlier conflict monitoring stage (N2) (Groom and Cragg, 2015). Second, P3 has been established as a robust and reliable electrophysiological marker of response inhibition, potentially offering greater consistency than N2 across studies and paradigms (Kropotov et al., 2011; Randall and Smith, 2011). Finally, research shows that long-term sport-specific training can enhance cognitive function and neural efficiency (Bonetti et al., 2025), with the P3 component serving as a sensitive measure of these adaptive neurocognitive changes (Wang et al., 2020; Prema et al., 2021). At its core, the Go/No-go paradigm operationalizes response inhibition by creating a conflict between the high-frequency Go responses and the occasional requirement to withhold a response on No-go trials (Gomez et al., 2007). Therefore, employing the Go/No-go paradigm to assess these mechanisms is well justified (Wright et al., 2014). Specifically, the P3 component is robustly elicited during successful No-go trials, serving as a direct neurophysiological index of inhibitory resource allocation. In this context, the amplitude of P3 reflects the intensity of cognitive engagement in the inhibitory process, while its latency marks the timing of this process relative to the stimulus (Picton, 1992).

In summary, while prior research has established the acute effects of single-session tDCS, the cumulative neurobehavioral impact of repeated tDCS in athletes remains underexplored, particularly using direct neural measures. To address this gap, this study employs a longitudinal, triple-blind, sham-controlled design to investigate whether a 4-week repeated tDCS protocol can induce sustainable enhancements in cognitive-motor performance in soccer players. In this exploratory study, we aim to investigate both the behavioral outcomes and the underlying neurophysiological mechanisms—specifically through modulation of the P3 component—that may mediate these potential improvements. Given the preliminary nature of repeated tDCS research in athletic populations, the present findings are intended to generate hypotheses and provide a foundation for future confirmatory trials. This approach allows us to move beyond verifying cumulative behavioral effects and toward explaining how repeated tDCS may optimize the neural efficiency of networks supporting response control in athletic populations. The findings are thus expected to provide a critical neurophysiological evidence base for advancing targeted, neuroscience-guided cognitive training protocols in sports science.

## 2 Materials and methods

### 2.1 Experimental subject

#### 2.1.1 Sample size estimation and justification

*A priori* sample size estimation was performed using G*Power software (version 3.1) (Faul et al., 2007). Based on previous literature (Brysbaert, 2019; Voss et al., 2010) and the present study design, a repeated-measures analysis of variance (ANOVA) model was selected. The significance level (*α*) was set at 0.05, and the desired statistical power (1 − *β*) was set at 0.80. Given that previous studies have reported medium effect sizes for differences in inhibitory control among athletes (Willcutt et al., 2005), a medium effect size (*Cohen’s f* = 0.3) was used for the calculation. This analysis indicated that a minimum total sample size of 30 participants was required. To account for potential sample attrition and enhance the robustness of results, the final sample size was increased to 36 participants. Although the sample size was determined based on *a priori* power analysis, the findings should be interpreted as preliminary due to the relatively modest sample and the exploratory nature of this repeated tDCS protocol in athletes.

#### 2.1.2 Participant recruitment and criteria

A total of 36 healthy male soccer players, all holding Chinese National Grade II Athlete certification or higher, were recruited for this study. All participants were right-handed to control for potential confounding effects of handedness on brain lateralization and tDCS-induced neuromodulation. They also had normal or corrected-to-normal vision. Individuals were excluded if they had a history of major surgery, neurological or psychiatric disorders, use of medications known to affect cognitive function, or prior participation in similar experiments within the past 6 months. Only male participants were enrolled to control for potential gender related confounding effects on the outcomes. Participants were recruited through convenience sampling from local university sports teams.

#### 2.1.3 Ethics approval and trial registration

This study was conducted in accordance with the principles of the Declaration of Helsinki. The study protocol received approval from the Ethics Committee of the School of Physical Education, Jiangxi Normal University (Approval No. IRB-JXNU-PEC-2025013). Written informed consent was obtained from all participants prior to their involvement in the study. As a pre-specified sub-study of the prospectively registered trial “The Effect of Different Training Methods on the Response Inhibition Ability of Athletes with Open Skills/Closed Skills: A Study Based on ERP” (Chinese Clinical Trial Registry, Registration No. ChiCTR2500109387), this investigation was specifically designed to validate the neurophysiological effects of tDCS under rigorously controlled conditions to minimize placebo effects.

### 2.2 Experimental grouping and design

This study employed a triple-blind, randomized, sham-controlled design. Using SPSS software (version 26.0), an independent researcher not involved in subsequent procedures generated the randomization sequence and assigned the 36 participants to one of three groups (*n* = 12 per group): an active-tDCS group, a sham-tDCS group, and a no-intervention control group. One-way ANOVA confirmed that the groups did not differ significantly in terms of age, height, body weight, or years of training experience (all *p* > 0.05), indicating well-balanced baseline characteristics (Table 1).

**Table 1:** One-way ANOVA of baseline characteristics across the three groups.

Group	Age (years)	Height (cm)	Weight (kg)	Training experience (years)
Active-tDCS (*n* = 12)	21.28 ± 2.25	175.50 ± 4.52	70.33 ± 7.13	5.91 ± 2.02
Sham-tDCS (*n* = 12)	21.36 ± 1.91	174.75 ± 3.93	69.58 ± 5.55	6.25 ± 1.71
No-intervention control (*n* = 12)	21.01 ± 1.52	175.41 ± 3.87	71.91 ± 3.98	6.08 ± 1.67
*F*	0.108	0.119	0.523	0.102
*p*	0.898	0.888	0.598	0.904

Data are presented as mean ± standard deviation (M ± SD).

**Figure 1:**
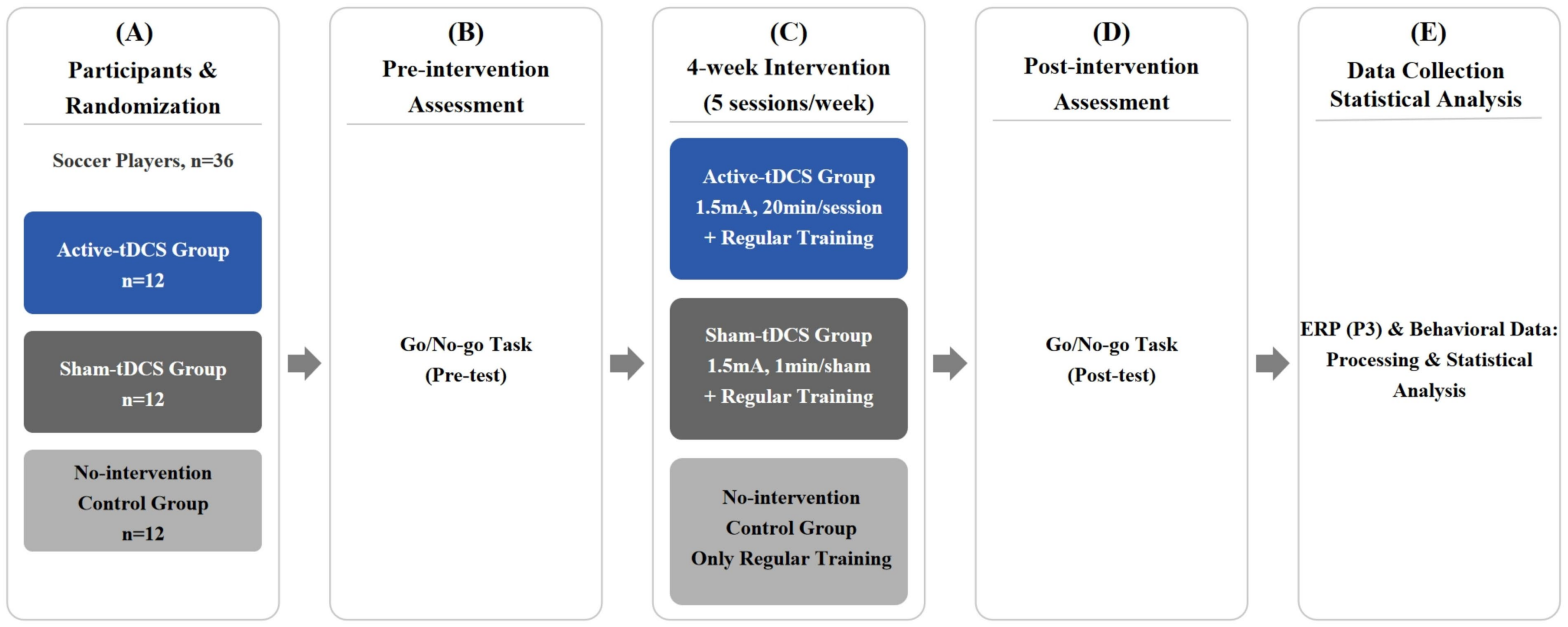
Flowchart of the experimental procedure. **(A)** Thirty-six soccer players were randomly assigned to three groups: active-tDCS, sham-tDCS, or no-intervention control (*n*=12 each). **(B)** All participants completed a Go/No-go task as a pre-test. **(C)** The active-tDCS group received 1.5 mA tDCS for 20 min per session, the sham-tDCS group received 1.5 mA for only 1 min, and the control group received no stimulation; all groups underwent regular training 5 sessions/week for 4 weeks. **(D)** A post-test Go/No-go task was administered to all participants. **(E)** Behavioral and ERP data were processed using MATLAB and statistically analyzed with SPSS 26.0.

Following randomization, all participants completed a pre-test using the Go/No-go response inhibition task. This was followed by a 4-week intervention period, during which the active-tDCS group received verum stimulation alongside their regular training, the sham-tDCS group received sham stimulation alongside regular training, and the no-intervention control group engaged in regular training only. A post-test was administered upon completion of the intervention. Within the 24 h preceding each testing session, participants were instructed to refrain from strenuous physical exercise and the consumption of caffeine or alcohol. A flowchart of the experimental procedure is presented in Figure 1.

### 2.3 tDCS intervention protocol

The tDCS intervention was administered using a DC-Stimulator PLUS device in an offline paradigm (i.e., stimulation was applied prior to cognitive testing). The stimulation parameters were selected based on established safety guidelines (Bikson et al., 2009; Bikson et al., 2016), previous studies (Borducchi et al., 2016), and the stimulation target was determined according to the research by Robertson and Marino (2016). The anode electrode (5 × 7 cm, 35 cm^2^) was positioned over the rDLPFC, corresponding to the F4 site according to the international 10–20 EEG system, with the cathode placed on the contralateral shoulder.

**Figure 2:**
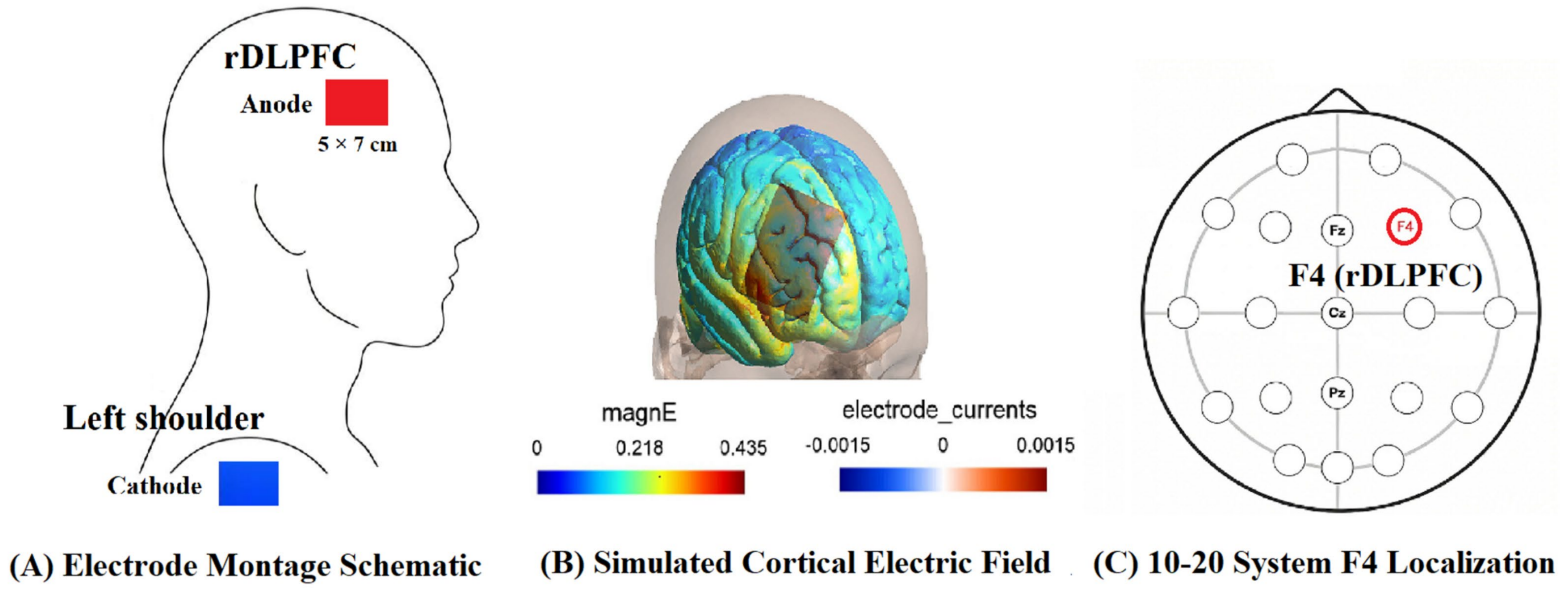
Electrode montage based on the 10–20 system and computational modeling of the induced electric field. **(A)** Schematic of the experimental electrode setup. The anode (red, 5 × 7 cm) was positioned over the right dorsolateral prefrontal cortex (rDLPFC). The cathode (blue, 5 × 7 cm) was placed on the contralateral shoulder. **(B)** Predicted electric field magnitude distribution on the cortical surface resulting from the montage shown in panel **(A)**, simulated using SimNIBS. The field is predominantly localized to the prefrontal region under the anode. The color bar indicates the field strength. **(C)** International 10–20 system for EEG electrode placement, providing the standard reference for locating the F4 site (anode position) used in this study.

To computationally model and visualize the electric field distribution induced by this electrode montage, a simulation was performed using SimNIBS 4.1.0 (Saturnino et al., 2019). The simulation was based on the standard head model “Ernie.” As the model’s anatomical scope does not include the shoulder, the extracephalic cathode was modeled as a large electrode at the lower posterior aspect of the head—a standard simplification employed in prior research to approximate the current return pathway (Masina et al., 2021). This computational approach, grounded in anatomically precise head modeling (Datta et al., 2009), provides a reliable estimate of the cortical electric field under the anode, as supported by empirical validation studies (Huang et al., 2017). The results of this simulation, detailing the electrode configuration, cortical field distribution, and axial cross-section, are presented in Figure 2.

In the active-tDCS group, stimulation was delivered at an intensity of 1.5 mA for a duration of 20 min, incorporating 30-s fade-in and fade-out periods at the beginning and end of the session. For the sham-tDCS group, all parameters were identical to the active-tDCS group, except the stimulation duration was reduced to 1 min (excluding fade-in/fade-out periods) to mimic the authentic sensory experience and ensure the integrity of the blinding procedure. To ensure the efficacy of the tDCS intervention while avoiding interference with the athletes’ regular training schedule, the tDCS sessions were conducted on training days. Furthermore, to minimize potential confounds from circadian rhythms and physical fatigue, all tDCS sessions were administered approximately 1 h before the participants’ regular training sessions. The intervention lasted for 4 weeks, with 5 sessions administered per week.

### 2.4 Blinding procedures

This study employed a triple-blind, randomized, sham-controlled design, ensuring that participants, experimenters, and the statistician remained unaware of group allocation throughout the trial.

#### 2.4.1 Allocation concealment

An independent researcher (randomization coordinator) who was not involved in participant recruitment, data collection, or analysis generated the randomization sequence using SPSS (version 26.0). This researcher prepared sequentially numbered, opaque, sealed envelopes. Each envelope contained the group assignment (active-tDCS, sham-tDCS, or no-intervention control) and the corresponding parameter instructions for the stimulation device.

#### 2.4.2 Handling of the no-intervention control group

To maintain blinding in the control group, all participants were informed prior to enrollment that the study involved three different “recovery or enhancement protocols”: electrical stimulation, sensory control stimulation, or focused rest. Participants in the no-intervention control group followed an identical visit schedule as the tDCS groups (5 sessions per week, approximately 30 min per session). During each visit, they were seated in the same laboratory room, wore a head cap (to mimic electrode placement), and were instructed to rest quietly. The same experimenter who stayed with the tDCS participants also stayed with the control participants, providing the same level of interaction (e.g., checking comfort, ensuring stillness) to equate the amount of attention received. Thus, control participants were not aware that they were in a “no-intervention” condition.

#### 2.4.3 Stimulation delivery procedure for tDCS groups

The specific intervention procedure for the active-tDCS and sham-tDCS groups was as follows: The randomization coordinator prepared sealed envelopes containing the parameter instructions. An experimenter (blind to group assignment) guided the participant into the laboratory and performed electrode placement according to the protocol described in Section 2.3. The experimenter then temporarily exited the room. An independent operator (not involved in any other aspect of the study) entered with the envelope, opened it, and configured the DC-Stimulator PLUS device in strict accordance with the enclosed instructions. After obscuring key information on the device screen and initiating the stimulation, the operator promptly departed. The experimenter re-entered the laboratory, remained with the participant throughout the 20-min intervention period, and completed standardized records. At the 20-min mark, the experimenter uniformly terminated the stimulation, concluding the session. For the sham-tDCS group, the device was programmed to deliver current only during the first minute (including 30-s fade-in and fade-out), while the experimenter and participant remained blind to this condition.

#### 2.4.4 Blinding of data analysis

The statistician received a fully de-identified dataset, with groups coded as A, B, and C, and remained blind to the coding scheme until the primary analyses were completed.

#### 2.4.5 Blinding assessment and adverse effects

Immediately after the post-test, all participants (including those in the no-intervention control group) were asked to guess which group they had been assigned to, with the following options: “active tDCS,” “sham tDCS,” or “unsure.” This allowed us to evaluate the success of participant blinding. Additionally, participants in the active-tDCS and sham-tDCS groups were asked to report any adverse sensations experienced during or after the stimulation sessions (e.g., tingling, itching, burning, headache, or discomfort). The frequency and severity of these symptoms were recorded for safety monitoring.

### 2.5 Response inhibition task and procedure

**Figure 3:**
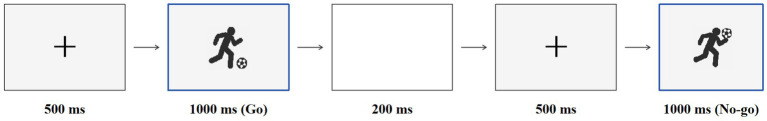
Go/No-go task flowchart.

A classic Go/No-go paradigm was employed to assess participants’ response inhibition skill. The task was programmed using E-Prime software (version 3.0). As depicted in Figure 3, the visual stimuli consisted of soccer scenario images: Go Stimulus: The image depicted a cartoon character dribbling the ball forward, representing a legal and positive action in soccer. Participants were instructed to press the “spacebar” as quickly as possible in response. No-go Stimulus: The image depicted a cartoon character committing a clear intentional handball foul, representing a prohibited action in soccer. Participants were required to inhibit their key press response and refrain from any action. Prior to the formal experiment, a practice block of 10 trials was administered, during which performance feedback was provided to ensure task comprehension. In the main experiment, each trial began with a fixation cross “+” presented at the center of the screen for 500 ms, followed by the random presentation of either a Go or No-go stimulus for 1,000 ms. A blank screen was then displayed for an inter-stimulus interval of 200 ms. The entire task comprised a total of 200 trials, distributed across two blocks (100 trials per block). The stimulus probability was set at 70% for Go trials (*n* = 140) and 30% for No-go trials (*n* = 60). A short rest period was allowed between the two blocks. The total duration of the experiment was approximately 8–10 min. Prior to the experiment, all participants confirmed that they had no prior experience with the computerized Go/No-go task used in this study.

### 2.6 Data processing and statistical analysis

#### 2.6.1 Behavioral data processing

Stimulus presentation and the collection of behavioral data were managed using E-Prime software (version 3.0). Data from practice trials and trials with an overall accuracy rate below 60% were excluded from the final analysis. Three primary behavioral measures were selected for analysis: Go reaction time (RT), Go trial accuracy (ACC), and No-go trial accuracy (ACC).

#### 2.6.2 ERP data processing

Electroencephalogram (EEG) signals were recorded using a 64-channel ERP acquisition system from Neuroscan Company. Electrodes were positioned according to the international 10–20 system. The signal resolution was set at 100 nV with a sampling rate of 1,000 Hz per channel. The FCz and AFz electrodes served as the reference and ground, respectively, and all electrode impedances were maintained below 10 kΩ. Offline data processing was performed using the EEGLAB toolbox running in a MATLAB environment. The EEG data processing pipeline included the following steps: (1) Removal of irrelevant electrodes (M2, HEOG, VEOG); (2) Re-referencing to the average of all channels; (3) Band-pass filtering between 0.1 Hz and 30 Hz; (4) Epoching from −200 ms to 1,100 ms relative to stimulus onset, followed by baseline correction using the −200 ms to 0 ms pre-stimulus interval; (5) Manual rejection of epochs containing amplitudes exceeding ±100 μV; (6) Independent component analysis to identify and remove artifacts associated with eye blinks, eye movements, and cardiac activity; (7) Averaging of artifact-free epochs to derive the ERP waveforms for each condition.

Although both the N2 and P3 components are associated with response inhibition, the present study focused its analysis on the P3 component for the following theoretical and methodological reasons. First, the primary aim was to investigate the cumulative, neuroplastic effects of a 4-week repeated tDCS protocol. The P3 component, reflecting later-stage cognitive evaluation and resource allocation, has been demonstrated to be more sensitive to sustained training and neuromodulation effects on processing efficiency compared to the earlier, conflict-monitoring related N2 component (Wang et al., 2020). Second, the observed behavioral effect of interest—reduction in Go RT—is more directly linked to the functional interpretation of P3 (stimulus evaluation and response decision speed) than to N2 (initial conflict detection). Finally, concentrating statistical power on a single, robust component (P3) provided a more precise test of our primary hypothesis within the current sample size. Therefore, the P3 mean amplitude and peak latency were selected as the primary neural metrics for this study.

Based on visual inspection of the grand-averaged ERP waveforms across all participants and in line with previous literature (Picton, 1992; Vázquez-Marrufo et al., 2013), the P3 component was quantified within a 300–450 ms post-stimulus time window. For statistical analysis, we extracted both the mean amplitude and the peak latency of the P3 component from the three midline electrode sites (Cz, CPz, Pz). This dual-metric approach is well-established in ERP research, as it allows for the concurrent assessment of the overall magnitude of neural engagement (mean amplitude) and the precise timing of the peak positive deflection (peak latency), thereby capturing complementary aspects of cognitive processing (Ruggeri et al., 2019).

To ensure reliable peak latency detection against high-frequency artifacts, we employed a rigorous two-step procedure combining automated and visual verification. First, the local positive maximum within the 300–450 ms window was identified automatically for each electrode. Subsequently, all detected peaks underwent independent visual inspection by two trained researchers. For a peak to be accepted, it had to satisfy three key criteria: it needed to correspond to a clear, monophasic positive deflection in the individual ERP waveform; its morphology had to be consistent with the P3 component observed in the grand-average data; and it must be clearly distinguishable from residual artifacts or high-frequency oscillations. Discrepancies between raters were resolved through consensus discussion. This combined approach significantly enhances the robustness of latency estimation and minimizes the risk of Type I errors from noise-driven spurious peaks.

#### 2.6.3 Statistical analysis

All statistical analyses were performed on behavioral and ERP measures. The normality of distribution for all dependent variables was first assessed using the Shapiro–Wilk test; all variables met the assumption of normality (*p* > 0.05), justifying the use of parametric repeated-measures analysis of variance (ANOVA). For behavioral measures, a two-way repeated-measures ANOVA was conducted with Group (active-tDCS, sham-tDCS, no-intervention control) and Time (pre-test, post-test) as factors on the following dependent variables: Go reaction time (RT), Go trial accuracy (ACC), and No-go trial ACC. For ERP measures, a three-way repeated-measures ANOVA was conducted with Group, Time, and Electrode (Cz, CPz, Pz) as factors on the P3 mean amplitude and peak latency. Separate analyses were performed for the Go and No-go conditions. Mauchly’s test was used to assess sphericity; when this assumption was violated, the Greenhouse–Geisser correction was applied. For significant main effects involving more than two levels, *post hoc* pairwise comparisons were performed using the Bonferroni correction. For significant interaction effects, simple effect analyses were conducted, followed by Bonferroni-corrected pairwise comparisons where appropriate. Partial eta-squared ( $\eta_{P}^{2}$ ) is reported as the measure of effect size. According to Cohen’s convention, $\eta_{P}^{2}$ values of 0.01, 0.06, and 0.14 were interpreted as small, medium, and large effects, respectively (Cohen, 2013). The significance level (*α*) was set at 0.05 for all tests. Data are presented as mean ± standard deviation (M ± SD). All analyses were conducted using SPSS (version 26.0).

## 3 Research results

### 3.1 Behavioral results

The results of the repeated-measures ANOVAs for all behavioral measures are summarized in Table 2, with statistically significant effects highlighted. For Go RT, a significant main effect of Time was observed, *F*(1,33) = 5.408, *p* = 0.026, $\eta_{P}^{2}$ = 0.141, indicating that the mean RT was significantly shorter during the post-test (415.87 ± 6.24 ms) compared to the pre-test (433.84 ± 6.05 ms). Furthermore, a significant Time × Group interaction was found for Go RT, *F*(2,33) = 3.483, *p* = 0.042, $\eta_{P}^{2}$ = 0.174. Simple effect analyses revealed that the active-tDCS group exhibited a significant reduction in RT from pre-test to post-test (*p* = 0.002, mean difference = 45.39 ms, 95% CI [18.16, 72.64]). In contrast, no significant changes were observed in the sham-tDCS group (*p* = 0.376) or the no-intervention control group (*p* = 0.796). Post-hoc pairwise comparisons with Bonferroni correction revealed no significant differences among the three groups at pre-test (all *p* > 0.05). At post-test, the active-tDCS group responded significantly faster than the no-intervention control group (*p* = 0.021, mean difference = 43.82 ms, 95% CI [5.37, 82.29]). However, the difference between the active-tDCS and sham-tDCS groups did not reach statistical significance (*p* = 0.148), nor did the difference between the sham-tDCS and no-intervention control groups (*p* = 0.785). No other main effects or interactions reached statistical significance.

**Table 2:** Results of the repeated-measures ANOVA for behavioral measures.

Measure	Effect	*df*	*F*	*p*	$\eta_{P}^{2}$
Go RT	Group	2,33	1.463	0.246	0.081
	Time	1,33	5.408	0.026*	0.141
	Time × Group	2,33	3.483	0.042*	0.174
Go ACC	Group	2,33	0.216	0.807	0.013
	Time	1,33	0.200	0.658	0.006
	Time × Group	2,33	1.400	0.261	0.078
No-go ACC	Group	2,33	0.855	0.435	0.049
	Time	1,33	0.091	0.764	0.003
	Time × Group	2,33	0.486	0.619	0.029

Bold values indicate significant main effects or interaction effects (*p < 0.05).

### 3.2 ERP results

ERP data from all 36 participants (active-tDCS group: *n* = 12; sham-tDCS group: *n* = 12; no-intervention control group: *n* = 12) were included in the analysis. The differences in P3 amplitude and latency during Go and No-go tasks before and after the intervention are reported below.

#### 3.2.1 P3 amplitude during go trials

To maximize statistical power for detecting the core effects of the tDCS intervention, we first averaged the P3 amplitude across the three electrode sites (Cz, CPz, Pz) and performed a two-way repeated-measures ANOVA with factors Group (active-tDCS, sham-tDCS, no-intervention control) and Time (pre-test, post-test). This analysis revealed a significant main effect of Time, *F*(1,33) = 23.721, *p* < 0.001, $\eta_{P}^{2}$ = 0.418, indicating that P3 amplitudes were overall larger during the post-test (3.16 ± 0.25 μV) compared to the pre-test (2.52 ± 0.27 μV).

More importantly, a critical Time × Group interaction was found, *F*(2,33) = 21.255, *p* < 0.001, $\eta_{P}^{2}$ = 0.563. Simple effect analyses (with Bonferroni correction) showed that only the active-tDCS group exhibited a significant increase in P3 amplitude from pre-test to post-test (*p* < 0.001, mean difference = 1.85 μV, 95% CI [1.388, 2.314]). In contrast, no significant changes were observed in the sham-tDCS group (*p* = 0.921) or the no-intervention control group (*p* = 0.842). Between-group comparisons indicated no significant differences in P3 amplitude at pre-test (all *p* > 0.05), confirming equivalent baseline levels. At post-test, the active-tDCS group demonstrated significantly larger P3 amplitudes than both the sham-tDCS group (*p* = 0.027, mean difference = 1.73 μV, 95% CI [0.162, 3.313]) and the no-intervention control group (*p* = 0.024, mean difference = 1.76 μV, 95% CI [0.190, 3.341]).

**Figure 4:**
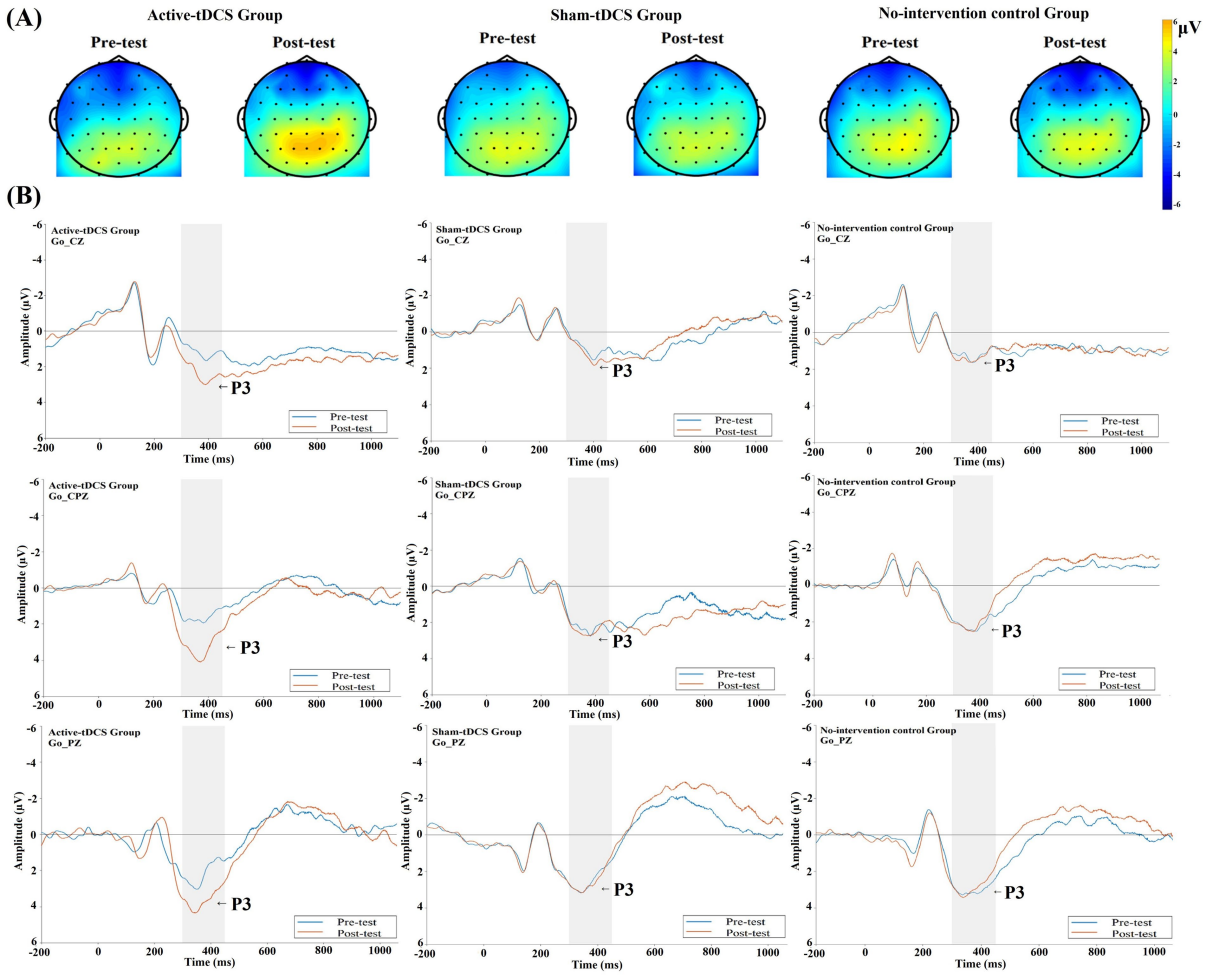
Go trials: **(A)** Topographic maps display the voltage distribution within the 300–450 ms time window for the active-tDCS, sham-tDCS, and no-intervention control groups, pre- and post-intervention. **(B)** Grand average waveforms at the Cz, CPz, and Pz electrodes. The blue and red lines represent pre-test and post-test recordings, respectively. The shaded area indicates the P3 time window (300–450 ms).

This interaction pattern is clearly visualized in Figure 4. The topographic maps (Figure 4A) show that at baseline, all three groups exhibited highly consistent spatial patterns of electrophysiological activity, with the positive voltage maximal near the centro-parietal region (Pz). Following the intervention, only the active-tDCS group displayed a marked enhancement in the intensity and spatial extent of this positivity in the parietal region, while the sham-tDCS and no-intervention control groups remained largely unchanged. The grand average waveforms (Figure 4B) visually demonstrate the increase in P3 amplitude for the active-tDCS group at electrodes Cz, CPz, and Pz.

To explore potential brain region specificity of the tDCS effect, we conducted a supplementary three-way repeated-measures ANOVA including the Electrode factor (Cz, CPz, Pz) (complete results in Table 3). This analysis reconfirmed the significant main effect of Time and the Time × Group interaction reported above. Additionally, the expected significant main effect of Electrode was observed, *F*(2,32) = 37.379, *p* < 0.001, $\eta_{P}^{2}$ = 0.700, with P3 amplitude increasing from Cz (1.70 ± 0.24 μV) to CPz (2.92 ± 0.30 μV) to Pz (3.90 ± 0.29 μV). However, none of the interactions involving the Electrode factor (including Electrode × Group, Electrode × Time, and the three-way interaction) reached statistical significance (all *p* > 0.05). *Post hoc* power analysis, based on the observed effect sizes for the non-significant interactions and our sample size, indicated insufficient power to detect small-to-medium effects. Therefore, this study did not yield evidence to support that the modulatory effect of tDCS on P3 amplitude during Go trials varies by brain region.

**Table 3:** Repeated-measures ANOVA results for P3 amplitude during Go trials.

Effect	*df*	*F*	*p*	$\eta_{P}^{2}$
Group	2,33	1.209	0.311	0.068
Electrode	2,32	37.379	<0.001*	0.700
Time	1,33	23.721	<0.001*	0.418
Time × Group	2,33	21.255	<0.001*	0.563
Electrode × Group	4,64	0.170	0.953	0.011
Electrode × Time	2,32	1.889	0.168	0.106
Electrode × Time × Group	4,64	1.599	0.185	0.091

Bold values indicate significant main effects or interaction effects (*p < 0.05).

#### 3.2.2 P3 latency during go trials

To maximize statistical power for detecting the core effects of the tDCS intervention on processing speed, we first averaged the P3 latency across the three electrode sites (Cz, CPz, Pz) and performed a two-way repeated-measures ANOVA with factors Group and Time. This analysis revealed a significant main effect of Time, *F*(1,33) = 48.606, *p* < 0.001, $\eta_{P}^{2}$ = 0.596, indicating that P3 latencies were overall shorter during the post-test (370.83 ± 3.06 ms) compared to the pre-test (376.96 ± 2.90 ms).

Critically, a significant Time × Group interaction was found, *F*(2,33) = 46.568, *p* < 0.001, $\eta_{P}^{2}$ = 0.738. Simple effect analyses (with Bonferroni correction) demonstrated that only the active-tDCS group exhibited a significant reduction in P3 latency from pre-test to post-test (*p* < 0.001, mean difference = 18.02 ms, 95% CI [14.93, 21.12]). In contrast, no significant changes were observed in the sham-tDCS group (*p* = 0.449) or the no-intervention control group (*p* = 0.323). Between-group comparisons indicated no significant differences in P3 latency either at pre-test (all *p* > 0.05) or at post-test (all *p* > 0.05).

This latency-shortening effect specific to active-tDCS is visually apparent in the grand average waveforms (Figure 4B). The peak of the P3 component for the active-tDCS group shows a clear leftward shift (indicating shorter latency) from pre-test to post-test, whereas the peaks for the sham-tDCS and no-intervention control groups remain relatively stable across sessions.

To explore potential topographical differences, a supplementary three-way ANOVA including the Electrode factor was conducted (complete results in Table 4). This analysis reconfirmed the significant main effects of Time and Electrode, and the Time × Group interaction. A significant main effect of Electrode was observed, *F*(2,32) = 46.685, *p* < 0.001, $\eta_{P}^{2}$ = 0.745, with P3 latency decreasing from Cz (396.31 ± 4.16 ms) to CPz (372.77 ± 3.26 ms) to Pz (352.59 ± 3.54 ms). However, none of the interactions involving the Electrode factor reached statistical significance (all *p* > 0.05). Similar to the P3 amplitude findings, *post hoc* power analysis suggested insufficient power to detect small-to-medium effects in these higher-order interactions for P3 latency. Therefore, this study did not yield evidence that the latency-shortening effect of tDCS varies by brain region during Go trials.

**Table 4:** Repeated-measures ANOVA results for P3 latency during Go trials.

Effect	*df*	*F*	*p*	$\eta_{P}^{2}$
Group	2,33	0.754	0.478	0.044
Electrode	2,32	46.685	<0.001*	0.745
Time	1,33	48.606	<0.001*	0.596
Time × Group	2,33	46.568	<0.001*	0.738
Electrode × Group	4,64	0.950	0.441	0.056
Electrode × Time	2,32	0.188	0.830	0.012
Electrode × Time × Group	4,64	2.309	0.067	0.126

Bold values indicate significant main effects or interaction effects (*p < 0.05).

#### 3.2.3 P3 amplitude during no-go trials

To maximize statistical power for detecting the core tDCS intervention effects, we first averaged the P3 amplitude across the three electrode sites (Cz, CPz, Pz) for No-go trials and performed a two-way repeated-measures ANOVA with factors Group and Time. This analysis revealed a significant main effect of Time, *F*(1,33) = 58.678, *p* < 0.001, $\eta_{P}^{2}$ = 0.640, indicating that P3 amplitudes were overall larger during the post-test (5.43 ± 0.32 μV) compared to the pre-test (4.54 ± 0.38 μV).

Crucially, a significant Time × Group interaction was found, *F*(2,33) = 56.394, *p* < 0.001, $\eta_{P}^{2}$ = 0.774. Simple effect analyses (Bonferroni-corrected) demonstrated that only the active-tDCS group showed a significant increase in P3 amplitude from pre-test to post-test (*p* < 0.001, mean difference = 2.62 μV, 95% CI [2.215, 3.030]). No significant changes were observed in the sham-tDCS group (*p* = 0.642) or the no-intervention control group (*p* = 0.777). Between-group comparisons confirmed equivalent baseline levels at pre-test (all *p* > 0.05). At post-test, the active-tDCS group exhibited significantly larger P3 amplitudes than both the sham-tDCS group (*p* = 0.011, mean difference = 2.43 μV, 95% CI [0.462, 4.412]) and the no-intervention control group (*p* = 0.007, mean difference = 2.56 μV, 95% CI [0.590, 4.540]).

**Figure 5:**
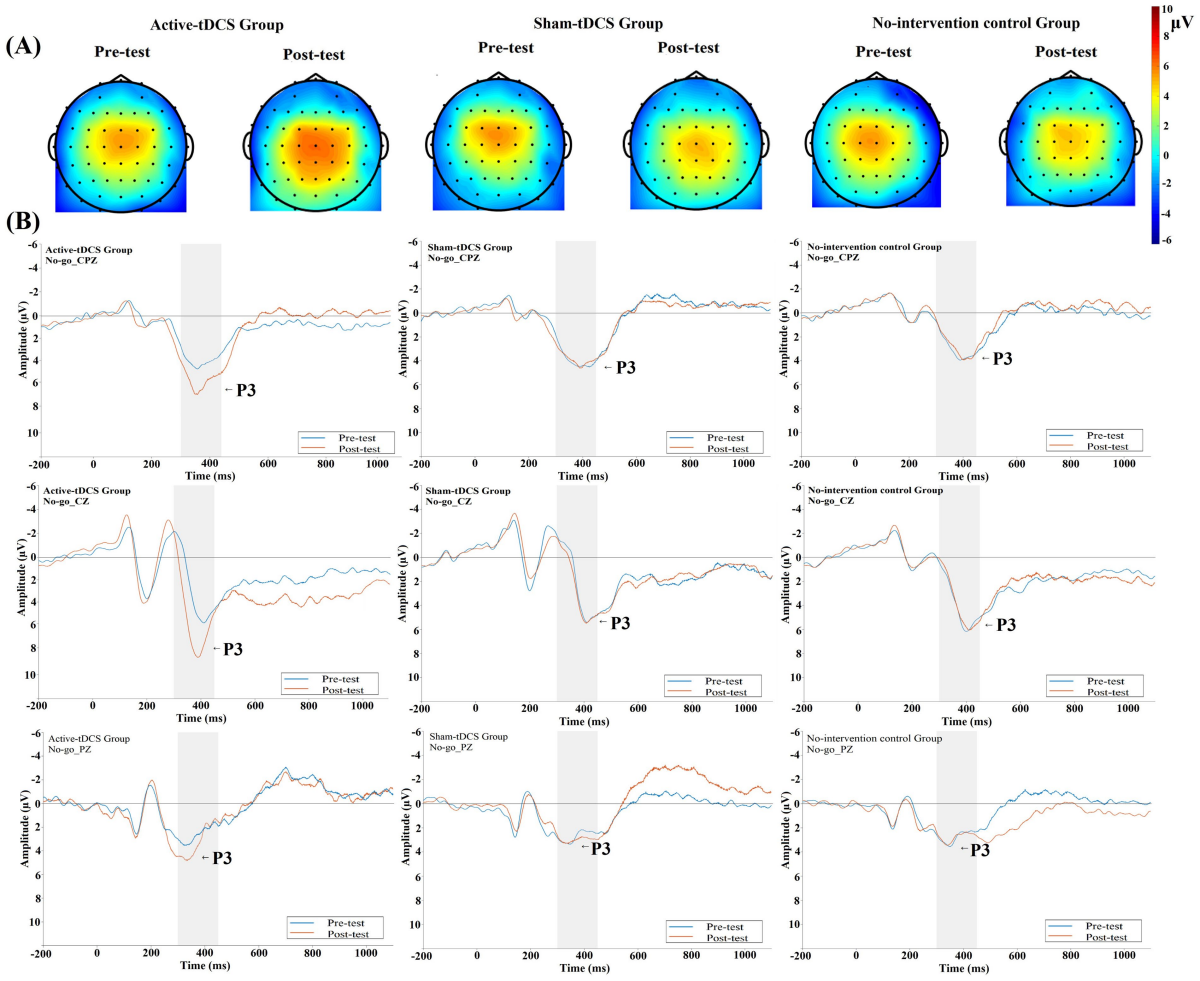
No-go trials: **(A)** Topographic maps display the voltage distribution within the 300–450 ms time window for the active-tDCS, sham-tDCS, and no-intervention control groups, pre- and post-intervention. **(B)** Grand average waveforms at the Cz, CPz, and Pz electrodes. The blue and red lines represent pre-test and post-test recordings, respectively. The shaded area indicates the P3 time window (300–450 ms).

This interaction is clearly visualized in Figure 5. Topographic maps (Figure 5A) show that at baseline, all groups exhibited consistent activity maximal near the central region (Cz). Following the intervention, only the active-tDCS group displayed a marked enhancement in the intensity and spatial extent of this central positivity. The grand average waveforms (Figure 5B) visually corroborate this amplitude increase.

To explore potential topographical specificity, a supplementary three-way ANOVA including the Electrode factor was conducted (complete results in Table 5). This analysis reconfirmed the significant main effects and the Time × Group interaction. A significant main effect of Electrode was observed, *F*(2,32) = 37.641, *p* < 0.001, $\eta_{P}^{2}$ = 0.702, with P3 amplitude decreasing from Cz (6.60 ± 0.42 μV) to CPz (4.63 ± 0.37 μV) to Pz (3.73 ± 0.33 μV), indicating a distinct anterior distribution for the No-go P3 compared to the Go P3. However, none of the interactions involving the Electrode factor reached statistical significance (all *p* > 0.05). Consistent with the Go trial findings, *post hoc* power analysis indicated insufficient power to detect small-to-medium effects in these higher-order interactions. Therefore, this study did not yield evidence that the tDCS-induced enhancement of No-go P3 amplitude varies by brain region.

**Table 5:** Repeated-measures ANOVA results for P3 amplitude during No-go trials.

Effect	*df*	*F*	*p*	$\eta_{P}^{2}$
Group	2,33	1.301	0.286	0.073
Electrode	2,32	37.641	<0.001*	0.702
Time	1,33	58.678	<0.001*	0.640
Time × Group	2,33	56.394	<0.001*	0.774
Electrode × Group	4,64	0.165	0.955	0.010
Electrode × Time	2,32	1.819	0.179	0.102
Electrode × Time × Group	4,64	1.384	0.249	0.080

Bold values indicate significant main effects or interaction effects (*p < 0.05).

#### 3.2.4 P3 latency during no-go trials

To maximize statistical power for detecting the core tDCS intervention effects on processing speed, we first averaged the P3 latency across the three electrode sites (Cz, CPz, Pz) for No-go trials and performed a two-way repeated-measures ANOVA with factors Group and Time. This analysis revealed a significant main effect of Time, *F*(1,33) = 21.142, *p* < 0.001, $\eta_{P}^{2}$ = 0.390, with overall shorter latencies at post-test (381.52 ± 3.02 ms) compared to pre-test (386.76 ± 3.23 ms).

Critically, a significant Time × Group interaction was also found, *F*(2,33) = 16.865, *p* < 0.001, $\eta_{P}^{2}$ = 0.505. Simple effect analyses (Bonferroni-corrected) confirmed that only the active-tDCS group exhibited a significant reduction in P3 latency from pre- to post-test (*p* < 0.001, mean difference = 14.58 ms, 95% CI [10.56, 18.60]). No significant changes were observed in the sham-tDCS (*p* = 0.978) or no-intervention control groups (*p* = 0.587). Between-group comparisons confirmed equivalent baseline levels at pre-test (all *p* > 0.05) and revealed no significant overall differences at post-test (all *p* > 0.05).

An exploratory three-way ANOVA including the Electrode factor was conducted to investigate potential topographical differences (complete results in Table 6). This analysis reconfirmed the significant main effects of Time and Electrode (*F*(2,32) = 89.086, *p* < 0.001, $\eta_{P}^{2}$ = 0.848), and the Time × Group interaction. A significant Electrode × Time interaction was also observed, *F*(2,32) = 12.187, *p* < 0.001, $\eta_{P}^{2}$ = 0.432, indicating that the pattern of P3 latency change across electrodes differed between pre- and post-test.

**Table 6:** Repeated-measures ANOVA results for P3 latency during No-go trials.

Effect	*df*	*F*	*p*	$\eta_{P}^{2}$
Group	2,33	0.868	0.429	0.050
Electrode	2,32	89.086	<0.001*	0.848
Time	1,33	21.142	<0.001*	0.390
Time × Group	2,33	16.865	<0.001*	0.505
Electrode × Group	4,64	0.620	0.650	0.037
Electrode × Time	2,32	12.187	<0.001*	0.432
Electrode × Time × Group	4,64	4.767	0.002*	0.230

Bold values indicate significant main effects or interaction effects (*p < 0.05).

Most importantly, a significant three-way Electrode × Time × Group interaction was found, *F*(4,64) = 4.767, *p* = 0.002, $\eta_{P}^{2}$ = 0.230. This indicates that the effect of the tDCS intervention on P3 shortening during No-go trials varied significantly across electrode sites. To unpack this complex interaction, separate two-way Time × Group repeated-measures ANOVAs were conducted for each electrode. At the Cz electrode, a significant Time × Group interaction was found, *F*(2,33) = 19.917, *p* < 0.001, $\eta_{P}^{2}$ = 0.547. The active-tDCS group showed a pronounced latency reduction (*p* < 0.001, mean difference = 32.00 ms, 95% CI [24.32, 39.67]), culminating in significantly shorter latency than the no-intervention control group at post-test (*p* = 0.019, mean difference = 25.66 ms, 95% CI [3.49, 47.84]). At the CPz electrode, a significant Time × Group interaction was found, *F*(2,33) = 4.711, *p* = 0.016, $\eta_{P}^{2}$ = 0.222. The active-tDCS group showed a pronounced latency reduction (*p* < 0.001, mean difference = 11.33 ms, 95% CI [5.49, 17.17]) and exhibited shorter latency than the sham-tDCS group at post-test (*p* = 0.033, mean difference = 20.16 ms, 95% CI [1.28, 39.04]). At the Pz electrode, the Time × Group interaction was not significant, *F*(2,33) = 0.682, *p* = 0.513, $\eta_{P}^{2}$ = 0.040.

This anteriorly graded pattern is visually corroborated in Figure 5B. The grand average waveforms show a clear leftward shift (shorter latency) of the P3 peak for the active-tDCS group at the Cz and CPz electrodes from pre- to post-test, while its latency at Pz, as well as all latencies in the sham-tDCS and no-intervention control groups across sites, remained stable.

### 3.3 Blinding and safety results

#### 3.3.1 Blinding assessment

After the post-test, all 36 participants completed the blinding questionnaire. In the active-tDCS group (*n* = 12), 5 participants (41.7%) correctly guessed their group assignment, 4 (33.3%) guessed incorrectly (sham), and 3 (25.0%) were unsure. In the sham-tDCS group (*n* = 12), 4 participants (33.3%) correctly guessed their group (sham), 5 (41.7%) guessed active, and 3 (25.0%) were unsure. In the no-intervention control group (*n* = 12), 3 participants (25.0%) believed they were in the active group, 4 (33.3%) believed they were in the sham group, and 5 (41.7%) were unsure. No participant in the control group reported believing they were in a “no-intervention” condition.

To quantify blinding success, Bang’s Blinding Index (Bang et al., 2004) was calculated for the active and sham groups. For the active-tDCS group, the index was 0.08 (95% CI [−0.40, 0.57]); for the sham-tDCS group, it was −0.08 (95% CI [−0.57, 0.40]). Both confidence intervals include zero, indicating no significant deviation from chance-level guessing and thus suggesting successful blinding. For the no-intervention control group, the index could not be computed due to the absence of a correct guess option, but the distribution of guesses (3 participants believed they were in the active group, 4 in the sham group, and 5 were unsure) indicates that participants were unaware of their true condition.

#### 3.3.2 Adverse effects

Mild itching or tingling sensations at the electrode site were reported by 4 participants in the active-tDCS group and 3 in the sham-tDCS group during the first few sessions. These sensations were transient and subsided shortly after the stimulation ended, with no recurrence in subsequent sessions. No participants reported severe discomfort, headache, or requested to discontinue the intervention. No adverse events were reported in the no-intervention control group.

## 4 Discussion

The present study sought to address the lack of longitudinal research on the cumulative neurobehavioral effects of repeated tDCS in athletes. By applying a 4-week tDCS protocol over the rDLPFC and employing ERP measures during a sport-relevant inhibitory control task, we demonstrated that active tDCS, compared to sham and no-intervention controls, led to improved behavioral response speed and concomitant neurophysiological optimization indexed by increased P3 amplitude and shortened P3 latency. This pattern of results provides preliminary, mechanistic evidence that repeated tDCS may enhance the efficiency of cognitive-motor processes in skilled athletes. However, given the exploratory nature of this study and the relatively small sample size, these findings should be interpreted with caution and require replication in larger cohorts.

### 4.1 Discussion of behavioral results

The behavioral findings suggest that tDCS may have primarily influenced response speed, as no significant beneficial effects were observed on response accuracy. Only the active-tDCS group showed a significant reduction in Go RT post-intervention, with their response speed becoming significantly faster than that of the no-intervention control group. While the direct comparison with the sham-tDCS group did not reach statistical significance, a trend toward improvement was noted. This pattern suggests that the enhancement in reaction speed may be attributable, at least in part, to the physiological effects of tDCS, rather than solely to non-specific factors such as the placebo effect or task practice (Price et al., 2008). This interpretation finds further support in the neurophysiological data (see below), tentatively indicating that repeated tDCS could provide cognitive benefits beyond conventional training and psychological expectation. For soccer, a sport that demands rapid decision-making, a millisecond-level improvement in reaction speed could be crucial (Knoop et al., 2013). However, it should be noted that reaction speed was measured in a laboratory task distinct from actual football performance; therefore, extrapolation to on-field performance should be made with caution.

The absence of improvement in response accuracy following tDCS intervention is noteworthy and likely attributable to a ceiling effect inherent in the experimental paradigm (McBee et al., 2010). Given that the participants were skilled soccer players, the cognitive demands of the standard Go/No-go task may have been insufficient to challenge their baseline inhibitory capacity, resulting in near-perfect accuracy rates at both pre- and post-test. The presence of this ceiling effect carries important implications for future research aiming to optimize cognitive training in athletes. To circumvent this limitation and better capture the full spectrum of tDCS effects, subsequent studies should employ paradigms with adaptive difficulty or increased cognitive load. For instance, integrating the Stop-Signal Task (SST) or introducing perceptual-motor conflict via Flanker tasks could provide a more sensitive measure of inhibitory control by pushing accuracy below ceiling.

### 4.2 Discussion of ERP results

The present study focused its electrophysiological analysis on the P3 component as the primary neural index of tDCS-induced changes in response inhibition. While the N2 component is a valid marker of early conflict monitoring (Patel and Azzam, 2005), several factors specific to our research questions and design justified this focused approach. First, our intervention employed a repeated tDCS protocol over 4 weeks, aiming to induce cumulative neuroplastic adaptations. The P3 component, reflecting later-stage cognitive evaluation and resource allocation (Rietdijk et al., 2014), has been shown to be particularly sensitive to sustained training and neuromodulation effects on processing efficiency, making it a theoretically more direct target for our longitudinal intervention than the earlier, more transient N2 (Wang et al., 2020). Second, the observed behavioral improvement—specifically reduced Go reaction time—aligns more closely with the functional interpretation of P3 (stimulus evaluation and response decision speed) than with N2 (initial conflict detection). The convergent findings of increased P3 amplitude and shortened latency provide a coherent neurophysiological explanation for the faster behavioral responses, thereby validating our component selection. Finally, concentrating our statistical power on a single, robust component (P3) allowed for a more precise and powerful test of our primary hypothesis within the current sample size, reducing the risk of Type I error associated with multiple comparisons across multiple ERP components.

The ERP results provide compelling neurophysiological evidence that aligns with and explains the observed behavioral improvement in response speed. To maximize statistical power while exploring topographic specificity, we employed a hierarchical analytical approach, first confirming overall tDCS effects via two-way ANOVAs (Group × Time) on data averaged across electrodes (Cz, CPz, Pz), then conducting supplementary three-way ANOVAs including the Electrode factor. This approach revealed that repeated tDCS specifically modulated the P3 component—a well-established index of attentional resource allocation (amplitude) and stimulus evaluation speed (latency) (Picton, 1992)—exclusively in the active-tDCS group.

The pattern of P3 modulation provides a neural basis for the behavioral facilitation. First, the increase in P3 amplitude was more pronounced during the No-go task than the Go task, suggesting that tDCS preferentially enhances neural circuits involved in inhibitory control (Simmonds et al., 2008). This enhanced resource allocation (indexed by larger P3 amplitude) likely supports faster resolution of response competition, thereby contributing to reduced Go reaction times (RT). Second, and more informatively, the shortening of P3 latency exhibited task-dependent topographic specificity. While significant latency reduction was observed in both tasks, a significant three-way interaction (Electrode × Time × Group) emerged only for No-go trials. Post-hoc analyses localized this effect primarily to the central Cz and CPz sites—regions overlying the sensorimotor and supplementary motor areas that are critical for motor inhibition and conflict monitoring (Durston et al., 2002)—with no significant change at the posterior Pz site associated with perceptual processing (Szczepanski et al., 2010). This indicates that tDCS did not induce a global acceleration of cognitive processing but rather precisely optimized processing speed within the fronto-central networks directly responsible for executing inhibitory commands.

This convergence of ERP findings suggests a coherent neurocognitive mechanism: repeated tDCS enhances the functional efficiency of the fronto-parietal inhibitory control network. The concomitant increase in resource mobilization (larger P3 amplitude) and acceleration of stimulus evaluation within motor-inhibition regions (shorter anterior P3 latency) likely work in tandem to enhance the brain’s capacity for rapid response selection and suppression (Hogeveen et al., 2016). This neural optimization offers a plausible mechanistic explanation for the observed reduction in Go RT: by augmenting the underlying neural infrastructure for cognitive control, tDCS may facilitate quicker overall response execution, even in a task primarily measuring speed. The absence of comparable amplitude or latency changes in both the sham-tDCS and no-intervention control groups reinforces the conclusion that these effects stem from genuine tDCS-induced neuromodulation rather than non-specific factors such as placebo or practice (Price et al., 2008).

Notably, the clearest neural signature—topographically specific latency shortening—was observed during No-go trials, whereas the behavioral benefit was captured in Go RT. This dissociation underscores that tDCS may primarily enhance the efficiency of the inhibitory control network, which in turn supports faster adaptive responding across contexts. Future studies employing paradigms that more directly measure the speed of inhibitory action (e.g., Stop-Signal Task) could help establish a more direct link between these neural changes and corresponding behavioral indices. Furthermore, while our hierarchical analysis confirmed robust main effects of tDCS, the study may have been underpowered to detect subtle spatial interactions in some comparisons (e.g., higher-order interactions in Go trials). Larger-sample studies are therefore warranted to fully characterize the topographic profile of tDCS effects across different cognitive demands.

It must be emphasized that the current conclusions are derived from controlled laboratory measures. A direct link to real-world football performance remains to be established, as this study did not incorporate sport-specific variables. Future research should, therefore, seek to bridge these gaps by integrating both more sensitive cognitive paradigms and ecologically valid metrics—such as video-based decision-making tasks or on-field performance indicators—to directly evaluate the translational impact of tDCS on athletic performance.

### 4.3 Reinterpreting the findings: proactive vs. reactive inhibition

The pattern of results—enhanced Go response speed alongside increased No-go P3 amplitude and shortened fronto-central P3 latency—suggests a potential dissociation between the processes supporting rapid action execution and those underlying inhibitory control. This dissociation can be fruitfully interpreted within the framework of proactive versus reactive inhibition (Aron, 2010; Braver, 2011). Proactive inhibition refers to the anticipatory maintenance of task goals to bias responding, whereas reactive inhibition is triggered by an imperative stop signal and reflects the actual suppression of motor output.

In the Go/No-go task, the high proportion of Go trials (70%) likely encouraged a proactive inhibitory set, facilitating rapid execution of the dominant Go response. The selective shortening of Go RT in the active-tDCS group suggests that repeated stimulation over the right DLPFC may have enhanced proactive control, potentially contributing to faster action initiation. Conversely, the robust modulation of the No-go P3—particularly the latency reduction at central electrodes (Cz, CPz) overlaying motor regions—indicates improved reactive inhibition efficiency. The topographic specificity (no change at parietal Pz) implies that tDCS may have preferentially accelerated motor-related inhibitory processes rather than early perceptual evaluation.

Taken together, these findings suggest that repeated tDCS may influence both proactive and reactive aspects of inhibitory control, offering a potential explanation for the observed behavioral and electrophysiological changes. However, this interpretation should be considered preliminary, as the current task was not specifically designed to dissociate these two processes. Future studies employing paradigms that explicitly manipulate proactive and reactive demands (e.g., conditional stop-signal task, AX-CPT) (Aron et al., 2007; Braver et al., 2001) are needed to directly test this hypothesis and clarify the specific contributions of prefrontal tDCS to each inhibitory subcomponent.

### 4.4 Study limitations and future directions

While this randomized controlled trial provides novel evidence for the efficacy of tDCS in enhancing response speed in athletes, several aspects warrant further discussion and refinement in future research. Importantly, given the exploratory nature of this repeated tDCS protocol in athletic populations and the relatively modest sample size, the present findings should be considered preliminary and interpreted with caution.

First, although the sample size is comparable to that of many neurostimulation studies, its modest scale may have limited statistical power for detecting subtle between-group effects, such as differences between the active- and sham-tDCS conditions. In addition, the all-male composition of the sample restricts the generalizability of the findings to female athletes and other sports populations. Future studies employing larger and more diverse samples, including female participants, are needed to verify and extend these findings and to examine potential sex-based differences in responsiveness to tDCS.

Second, the link between the observed neurophysiological changes (P3 modulation) and behavioral outcomes (Go RT) should be interpreted with caution. Although the active-tDCS group showed specific neural enhancements concomitant with improved reaction speed, the direct behavioral difference between active and sham stimulation did not reach statistical significance. This indicates that the neural plasticity induced by tDCS may not have fully translated into a robust between-group behavioral advantage within the current paradigm. Future research should employ behavioral tasks more sensitive to the speed of inhibitory action, such as the Stop-Signal Task, to establish a more direct and compelling link between neuromodulation and the specific behavioral gain observed here—namely, improved response speed.

Beyond considerations of task sensitivity, a related methodological consideration lies in the analysis of the neural data itself. The use of a fixed 300–450 ms time window for P3 analysis, while facilitating standardized group-level comparisons and alignment with prior studies (Picton, 1992; Vázquez-Marrufo et al., 2013), may not fully account for individual differences in processing speed or condition-specific latency variations. Recent methodological advancements advocating for data-driven, individualized window determination (Mahini et al., 2024) offer promising avenues for future research to capture such temporal nuances more precisely, potentially revealing tighter correlations between neuromodulation effects, neural activity, and behavior.

Third, while the Go/No-go task, administered in a laboratory setting, is a well-validated measure of response inhibition, it may not fully capture the complex, dynamic demands of response inhibition during actual soccer match play. Future research could employ more ecologically valid paradigms, such as virtual reality simulations of competitive scenarios.

Fourth, the tDCS parameters and stimulation target in this study were selected based on prior literature (Bikson et al., 2016; Borducchi et al., 2016). However, the optimal stimulation protocol for athletic populations remains to be fully elucidated. Future work should explore parametric variations in intensity, duration, and stimulation site.

Fifth, this study assessed only the immediate “offline” benefits of repeated tDCS under well-controlled laboratory conditions. It did not evaluate the persistence of these effects beyond the post-intervention period or their transfer to real-world performance contexts, which represents a key limitation for translating laboratory findings into practical athletic enhancement. Therefore, future investigations should examine the durability of tDCS-induced improvements and explore the synergistic effects of combining tDCS with sport-specific training (Steinberg et al., 2019), designing integrated intervention protocols that closely mimic real-game environments. Additionally, future studies should include both male and female athletes to enable sex-stratified or sex-by-intervention analyses, as sex may moderate cognitive-behavioral responses to neuromodulation.

Finally, this study implemented a repeated tDCS intervention and confirmed its efficacy compared to no intervention and sham tDCS. However, the current design cannot distinguish whether the behavioral improvements stem from the cumulative neuroplasticity induced by long-term tDCS or the acute effects of the final stimulation session. Future studies could introduce an acute intervention control group for a direct comparison, allowing for a precise dissection of the cumulative versus acute effects of tDCS.

In summary, the present findings should be viewed as hypothesis-generating rather than confirmatory. Replication in larger, more diverse samples—including female athletes and individuals from different sports backgrounds—is essential before definitive conclusions can be drawn regarding the efficacy of repeated tDCS for enhancing inhibitory control in athletic populations.

## Data Availability

The data analyzed in this study is subject to the following licenses/restrictions: we wish to note that the raw data from this study are subject to a confidentiality agreement with our participants and are part of an ongoing research project. Therefore, the data are available upon reasonable request rather than in a public repository, as detailed in our Data Availability Statement. Access to the dataset is restricted and may only be granted upon reasonable request and with permission from the corresponding author or institution. Requests to access these datasets should be directed to Kaihao Chen, kevinchen9227@gmail.com.
